# The Implementation of DNA Methylation Profiling into a Multistep Diagnostic Process in Pediatric Neuropathology: A 2-Year Real-World Experience by the French Neuropathology Network

**DOI:** 10.3390/cancers13061377

**Published:** 2021-03-18

**Authors:** Melanie Pages, Emmanuelle Uro-Coste, Carole Colin, David Meyronet, Guillaume Gauchotte, Claude-Alain Maurage, Audrey Rousseau, Catherine Godfraind, Karima Mokhtari, Karen Silva, Dominique Figarella-Branger, Pascale Varlet, on behalf of the RENOCLIP-LOC Network

**Affiliations:** 1 GHU-Paris—Sainte-Anne Hospital, Paris University, 75014 Paris, France; 2 Département de Pathologie, CHU Toulouse, 31000 Toulouse, France; uro-coste.e@chu-toulouse.fr; 3 INSERM UMR 1037, Cancer Reaserch Center of Toulouse (CRCT), 31000 Toulouse, France; 4 Département d’Anatomie et Cytologie Pathologiques, Toulouse III University-Paul Sabatier, 31000 Toulouse, France; 5 Aix-Marseille University, CNRS, INP, Inst Neurophysiopathol, 13007 Marseille, France; carole.colin@univ-amu.fr (C.C.); DominiqueFrance.FIGARELLA@ap-hm.fr (D.F.-B.); 6 Groupe Hospitalier Est, Département de Neuropathologie, Hospices Civils de Lyon, 69500 Bron, France; david.meyronet@chu-lyon.fr (D.M.); karen.silva@chu-lyon.fr (K.S.); 7 Département de Pathologie, Hôpital Central, CHU, 54000 Nancy, France; guillaume.gauchotte@univ-lorraine.fr; 8 Service Anatomie Pathologique, Pôle Pathologie Biologique, CHU Lille, 59000 Lille, France; ca-maurage@chru-lille.fr; 9 Département de Pathologie Cellulaire et Tissulaire, CHU d’Angers, 49100 Angers, France; aurousseau@chu-angers.fr; 10 CRCINA Université de Nantes-Université d’Angers, 49100 Angers, France; 11 CHU Clermont-Ferrand, Service d’Anatomopathologie, 63003 Clermont-Ferrand, France; cgodfraind@chu-clermontferrand.fr; 12 Département de Neuropathologie Raymond Escourolle, Groupe Hospitalier Pitié-Salpêtrière, AP-HP, 75013 Paris, France; karima.mokhtari@aphp.fr; 13 APHM, CHU Timone, Service d’Anatomie Pathologique et de Neuropathologie, 13005 Marseille, France

**Keywords:** DNA methylation, pediatric CNS tumors, tumor classification, copy-number variation, integrated diagnosis, molecular pathology

## Abstract

**Simple Summary:**

Supported by the “easy-to-use” and free DKFZ central nervous system (CNS) tumor classification web tool, DNA methylation profiling is a method changing the routine diagnostic practice in neuro-oncology. This work depicts a real-world practice experience by the French neuropathology network of incorporating DNA methylation profiling into the diagnostic process of challenging pediatric CNS tumors. After two rounds of histopathological review by neuropathology experts—including morphology, neuroimaging, immunohistochemistry, panel sequencing and FISH—62 tumors still presenting diagnostic uncertainty were selected for DNA methylation profiling. Using the DKFZ “classifier” and combining all additional information obtained from DNA methylation array, we observed significant diagnostic refinements and amendments. DNA methylation was successful in a significant number of cases (71%) despite the complex specificities of the cohort. Our study evaluates how DNA methylation testing would impact diagnosis and presents illustrative and representative cases.

**Abstract:**

DNA methylation profiling has recently emerged as a powerful tool to help establish diagnosis in neuro-oncology. Here we present our national diagnostic strategy as the French neuropathology network (RENOCLIP-LOC) and our current approach of integrating DNA methylation profiling into our multistep diagnostic process for challenging pediatric CNS tumors. The tumors with diagnostic uncertainty were prospectively selected for DNA methylation after two rounds of review by neuropathology experts. We first integrated the classifier score into the histopathological findings. Subsequent analyses using t-SNE (t-Distributed Stochastic Neighbor Embedding) representation were performed. An additional step consisted of analyzing copy-number variation data (CNV). Finally, we combined all data to establish diagnoses and evaluated the impact of DNA methylation profiling on diagnostic and grading changes that would affect patient management. Over two years, 62 pediatric tumors were profiled. (1) Integrating the classifier score to the histopathological findings impacted the diagnosis in 33 cases (53%). (2) t-SNE analysis provided arguments for diagnosis in 26/35 cases with calibrated scores <0.84 (74.3%). (3) CNV investigations also evidenced alterations used for diagnosis and prognostication. (4) A diagnosis was finally established for 44 tumors (71%). Our results support the use of DNA methylation for challenging pediatric tumors. We demonstrated how additional methylation-based analyses complement the classifier score to support conventional histopathological diagnosis.

## 1. Introduction

The current WHO classification of CNS (central nervous system) tumors, which encompasses more than 150 histologically and/or molecularly defined entities, mirrors the wide range of distinct groups and subgroups of CNS tumors that have recently emerged, particularly in pediatrics [1]. Indeed, the recent molecular findings have led to refining the classification by introducing newly described entities as well as subdividing morphology-based entities into molecular subgroups, resulting in challenges for routine diagnostic practice [1,2,3,4,5,6,7,8]. In parallel to these findings, the recent technological advances made have encouraged the integration of molecular testing, alongside morphological examination for minimizing inter-observer variability in histopathological diagnosis. Because limiting diagnostic uncertainty is of crucial importance for better prognostication and for determining optimal treatment, the identification of all these morpho-molecular entities requires developing the right tools to assist decision-making in clinical practice, within a standardized diagnostic process. Recently, a genome-wide methylation profiling-based classification developed by the Heidelberg group has emerged as a robust powerful tool to help establish diagnosis in neuro-oncology [9,10,11,12,13]. Therefore, in addition to genomic and transcriptomic approaches, incorporating DNA methylation profiling into routine testing can enable a significant step toward improving diagnostic accuracy and reproducibility and help resolve histopathological diagnostic uncertainties. In that sense, the Heidelberg group made this approach applicable in a routine diagnostic setting by developing a free web tool accessible to everyone everywhere that provides an analysis report based on their DNA methylation-based CNS tumor “classifier” [9,10].

Here, we describe the French neuropathology network’s 2-year experience using this approach for pediatric CNS tumors that are deemed histologically diagnostically difficult and how we integrate the methylation data into the morpho-molecular diagnosis. We provide an overview of our scheme layered diagnostic process and focus on some illustrative and representative cases. We also evaluate how this approach of refining diagnosis and tumor grade can influence patient management.

## 2. Materials and Methods

### 2.1. Patients and Sample Preparation

All tumors profiled were selected at a national tumor board meeting held every month (or twice a month by virtual conference since COVID-19 has prevented “in-person” meetings), bringing together the neuropathology experts from every region of France who present any challenging cases they received from the pathology centers of their respective region. Only pediatric tumors were eligible for DNA methylation profiling and restricted selection criteria were: lack of consensus on the diagnosis, conflicting morphological and/or molecular findings, non-informative molecular testing or other confusing aspects. Three cases (#2, #16, #34) have been previously reported [14,15,16].

Area of representative tumor was selected from hematoxylin-phloxin-saffron stained sections and tumor cell content was estimated for each sample. Macrodissection was performed in some cases to enrich tumor cell content or to exclude hemorrhage, calcification or poor tissue quality area. The selected areas were scraped from three to six serial 10-μm-thick sections. When an insufficient DNA amount was extracted, additional sections were utilized. Post-scraping hematoxylin-phloxin-saffron stained sections were systematically examined to ensure sufficient tumor cell content.

### 2.2. DNA Extraction and Methylation Analysis

Technical steps were performed on the Integragen platform (Integragen Genomics, Evry, France). The DNA extraction was performed using the QIAamp DNA FFPE Tissue Kit and the Qiacube (QIAGEN, Hilden, Germany) according to the manufacturer’s instructions. Extracted DNA (250–500 ng) was bisulfite-converted using a Zymo EZ DNA methylation kit and ZR-96 DNA Clean and Concentrator-5 (Zymo Research, Irvine, CA, USA). Bisulfite DNA was processed using the Illumina Infinium HD FFPE DNA Restore kit and Infinium FFPE QC kit (Illumina, San Diego, CA, USA). The standard quality controls confirmed DNA quantity/quality and bisulfite conversion. The DNA was then processed using the Illumina Infinium HumanMethylation EPIC Bead-Chip array (Illumina, San Diego, CA, USA) according to the manufacturer’s instructions. The iScan control software was used to generate raw data files from the BeadChip in IDAT format, which were analyzed using GenomeStudio version 2.0 (Illumina, San Diego, CA, USA) and checked for quality measures according to the manufacturer’s instructions.

### 2.3. Data Processing

The .idat files were uploaded to the online CNS tumor DNA methylation classifier at https://www.molecularneuropathology.org (accessed on 17 March 2021) (v11b4) and a report for every tumor was generated, providing prediction scores for methylation classes and chromosomal copy-number plots. The calibrated scores were integrated into the histopathological findings according to the recommendations from Capper et al. [10].

Additional analyses were performed in R studio (v4.0.2). Raw signal intensities were obtained from .idat files using the minfi Bioconductor R package (v1.34.0). Background correction and dye-bias correction were performed on each sample. A correction for the type of specimen (FFPE or frozen) was performed with the removeBatchEffect function (limma R package v3.44.3). Filtering criteria of probes were removal of probes targeting X or Y chromosomes, removal of probes containing single nucleotide polymorphisms and probes not included in the EPIC array.

t-SNE (t-Distributed Stochastic Neighbor Embedding) was performed using the Rtsne package (v0.15). We selected the most variable probes for t-SNE (SD > 0.25) with parameters theta = 0, pca = TRUE, max_iter = 2500, perplexity = 10, based on the method described by Capper et al. [9]. A total of 675 CNS tumors corresponding to 53 methylation classes from the Heidelberg reference cohort were included. Tumors of our cohort with a calibrated score <0.84 were included in this analysis.

Chromosomal copy-number variations (CNV) were analyzed using plots generated by the MNP website as well as by generating additional plots using the conumee R package (v1.22.0). Focal CNVs observed on the plots were investigated using the segmented files and IGV visualization. All CNV plots were also checked for noise.

All other plots and graphs were generated using the ggplot2 (v3.3.2) package in R studio (v4.0.2).

## 3. Results

### 3.1. Workflow

Prior to reporting our experience of incorporating DNA methylation profiling in our diagnostic process, we briefly present the organization of the French neuropathology network (RENOCLIP-LOC Réseau de Neuro-Oncologie Clinico-pathologique pour les cancers rares du SNC) whose primary purpose is to jointly analyze difficult adult and pediatric CNS tumors, facilitate access to molecular biology platforms and harmonize the histopathological diagnosis of CNS tumors. This multistep organization enabling a second reading by experts at regional meetings and if necessary, a third reading at national case conferences, led by regional and national experts, is described in Figure S1. For every tumor discussed at a regional and/or national case conference, a report summarizing all results and a conclusion is provided to all practitioners involved in the patient management. DNA methylation profiling is only proposed as a last resort after a collegial decision is made by the national experts (bi-monthly virtual meeting) when consensus on the diagnosis has not been achieved because of conflicting morphological and/or molecular findings, non-informative molecular testing or other confusing aspects. Therefore, our work investigates the added value of DNA methylation profiling for diagnosis only within a group of particularly challenging pediatric CNS tumors after meticulous selection by nine neuropathology experts.

### 3.2. Integration of Data from the “Classifier”

DNA methylation profiling was introduced into our panel of diagnostic molecular testing in 2018. Between October 2018 and August 2020, a total of 1860 tumors were reviewed at RENOCLIP-LOC tumor board discussions, including 589 pediatric cases, of which 172 were reviewed at a national case conference. Seventy-five pediatric cases were referred by the experts for a diagnostic DNA methylation array. In the first step, DNA extraction was performed from formalin-fixed paraffin-embedded (FFPE) tissues, after tumor content evaluation. The tumor content median was 80% (range 30–100%). To optimize the tumor content or to exclude hemorrhage/calcification/poor quality tissue areas, a macrodissection was performed in 10 cases (16%). For 48 samples (77%), between 3 and 6 consecutive tissue sections of 10 μm thickness were easily sufficient to yield above our minimum input threshold of 250 ng of DNA. The median DNA yield was 1047 ng (range 262–6620 ng). DNA was extracted from frozen tissue for 3 tumors with a limited amount of available FFPE tissue. Finally, 62 tumors with sufficient quality/quantity of DNA were submitted to genome-wide DNA methylation profiling. The mean turnaround time between the DNA extraction and submission to the “classifier” was 25 days. The cohort of profiled tumors included 24 low-grade gliomas/glioneuronal tumors, 10 high-grade gliomas/glioneuronal tumors, 8 ependymal tumors, 16 embryonal tumors, 1 plexus choroid tumor and 3 unclassified tumors. Further clinical and histopathological information is detailed in Table S1.

**Figure 1:**
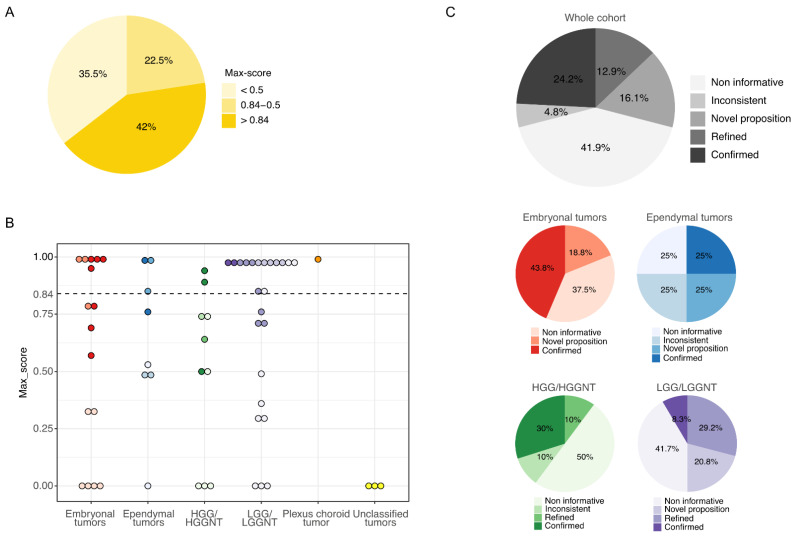
Results from the “classifier” and integration to the morpho-molecular data. (**A**) Proportion of the 62 tumors assigned in a methylation class with a calibrated max-score higher than 0.84 or between 0.5 and 0.84 or lower than 0.5. (**B**) Distribution of the calibrated max-scores in each category of the 62 challenging CNS (central nervous system) pediatric tumors. The color intensity of the dots reflects the integration of the classifier scores to the morpho-molecular data as depicted in 1C. The dotted line represents the threshold of high-confidence according to the recommendations from Capper et al. [10]. (**C**) Integration of the classifier scores to the morpho-molecular data of the whole cohort (top panel) and for each category of tumors (bottom panel). HGG/HGGNT = high-grade glial/glioneuronal tumor. LGG/LGGNT = low-grade glial/glioneuronal tumor.

We integrated the classifier score results with the histopathological findings according to the recommendations from Capper et al. [10] and calibrated scores between 0.84 and 0.3 were considered potentially useful but integrated with caution. A calibrated max-score higher than 0.84 was assigned in 26 of the 62 cases (42%). A max-score between 0.5 and 0.84 was obtained in 14 tumors (22.5%). A low max-score (<0.5) was assigned in 22 tumors (35.5%) (Figure 1A). A calibrated max-score higher than 0.84 was assigned in 6 of the 16 embryonal tumors (37.5%), in 3 of the 8 ependymal tumors (37.5%), in 2 of the 10 high-grade glial/glioneuronal tumors (20%) and in 14 of the 24 low-grade glial/glioneuronal tumors (50%). No methylation class was assigned to the three unclassified tumors (Figure 1B). The lowest scores were observed across all types of tumors and irrespective of DNA yield and tumor tissue quality. When integrating the “classifier” scores into the histopathological data, the initially proposed diagnosis was confirmed in 15 cases (24.2%) while DNA methylation profiling enabled us to precisely confirm 8 diagnoses (12.9%). In 10 cases (16.1%), a novel diagnostic hypothesis was proposed, leading to amendment of the final diagnosis. The “classifier” results were inconsistent or non-informative for, respectively, 3 (4.8%) and 26 (41.9%) tumors when integrating clinical, radiological and histopathological findings (Figure 1C). Within the embryonal tumor group, the initial diagnosis was confirmed in 7 cases (43.8%), the score suggested a novel diagnostic proposition in 3 cases (18.8%) and was non-informative in 6 cases (37.5%). Within the ependymal tumor group, the initial diagnosis was confirmed in 2 cases (25%), the score suggested a novel diagnostic proposition in 2 cases (25%), was inconsistent in 2 cases (25%) and was non-informative in 2 cases (25%). Within the high-grade glial/glioneuronal tumor group, the initial diagnosis was confirmed in 3 cases (30%) and refined in 1 case (10%), the score was inconsistent in one case (10%) and non-informative in 5 cases (50%). Within the low-grade glial/glioneuronal tumor group, the initial diagnosis was confirmed in 2 cases (8.3%) and refined in 7 cases (29.2%), the score suggested a novel diagnostic proposition in 5 cases (20.8%) and the score was non-informative in 10 cases (41.7%) (Figure 1C and Table S1).

We present two examples of representative cases with a novel diagnostic proposition:

Case #3

A 5-year-old female patient presented with an intraventricular (third ventricle) mass, diagnosed as low-grade glioma showing no particular morphological feature that could orientate the histopathological diagnosis to a subtype of pediatric low-grade glioma. The molecular testing performed did not permit the detection of any driver event, particularly in the MAPK pathway (Table S1). DNA methylation profiling was proposed given the uncertainty in the diagnosis and grading of this intraventricular glial tumor. The “classifier” assigned the tumor to the LGG, MYB/MYBL methylation class with a calibrated max-score of 0.99. This result was supported by the presence of a focal deletion at the position 8q13 on the CNV plot suggesting a *MYBL1* rearrangement, subsequently confirmed by RNA sequencing that identified a *MYBL1:CTB118P15.2* fusion. We thereby concluded with the integrated diagnosis of an intraventricular low-grade glioma with *MYBL1* fusion.

*Case #12*

A 5-year-old female patient presented with a spinal lesion for which a diagnosis of pilocytic astrocytoma *versus* spinal location of a rosette-forming glioneuronal tumor was proposed. Nevertheless, the molecular testing performed did not detect any *BRAF* or *FGFR1* disruption (Table S1). Due to the unclear differential diagnosis, a DNA methylation profiling was proposed. The tumor matched the diffuse leptomeningeal glioneuronal tumor (DLGNT) methylation class with a calibrated max-score of 0.98. The CNV analysis demonstrated several copy-number changes including 1p loss and 1q gain as well as focal deletions in 3p and 6q. RNA sequencing was performed and detected a *QKI:RAF1* fusion. We thereby concluded with the integrated diagnosis of a DLGNT with *QKI:RAF1* fusion.

### 3.3. Inference from t-SNE Representation

**Figure 2:**
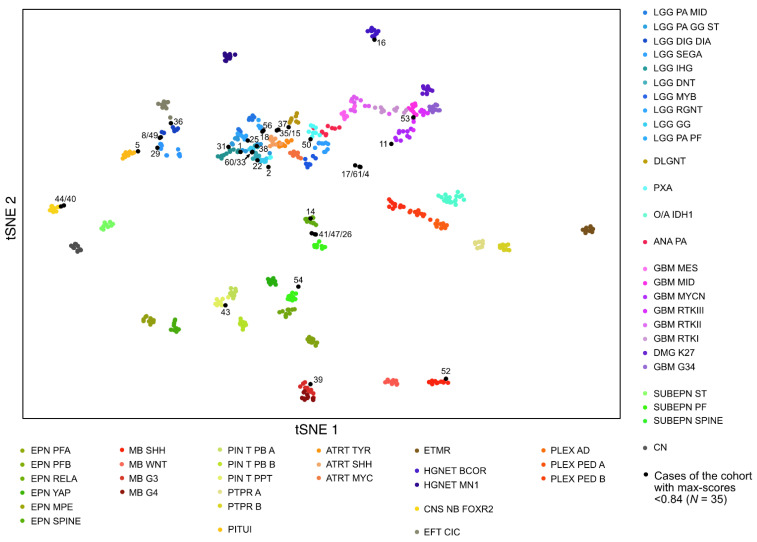
Methylation-based t-SNE (t-Distributed Stochastic Neighbor Embedding) distribution. The 35 tumors with a calibrated score <0.84 were compared with 675 reference samples from the German Cancer Research Center (DKFZ) cohort belonging to 53 methylation classes (colored dots). The 35 cases of this study are indicated as black dots and are shown by their ID number (see Table S1 for details on each case). Tumors that clustered within a reference group were considered belonging to the corresponding class if morpho-molecular data were consistent with the result.

To further explore the non-elucidated cases, we performed a t-SNE analysis including all cases with a max-score <0.84 (with the exception of one case with a max-score of 0.75 for cerebellum hemisphere), which represented 35 cases (22 non informative, 3 inconsistent, 1 novel proposition, 5 confirmed, 4 refined) (Figure 2). The analysis was contributive for 26 tumors (74.3%) which obviously clustered with a defined cluster (Table S1), including 10 cases with a calibrated max-score <0.3. Among these 26 tumors, the results of the t-SNE analysis were aligned with the calibrated max-score for 13 cases. The results of the t-SNE analysis were consistent with the morpho-molecular data for 16/26 cases and were considered for the integrated diagnosis (Table S1).

**Figure 3:**
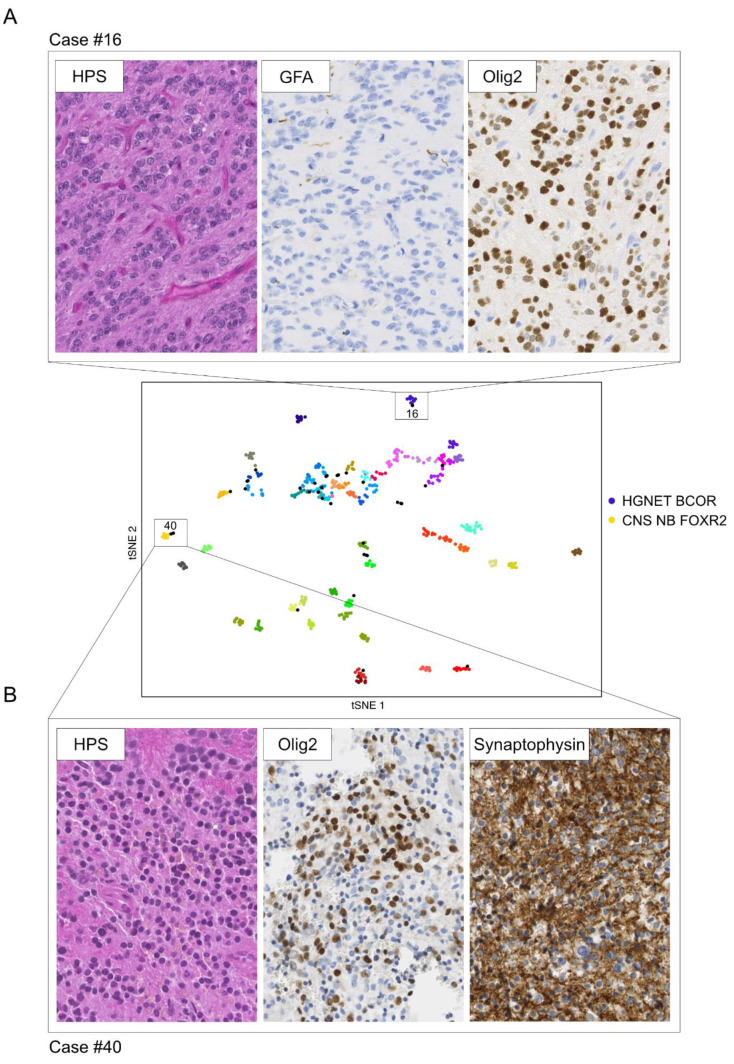
Examples illustrating the impact of the t-SNE analysis on establishing diagnosis. (**A**) Hematoxylin-phloxin-saffron stained sections of a compact ependymoma-like tumor with branching vessels showing no GFAP expression but Olig2 expression (case #16). The calibrated max-score was 0.57 for HGNET BCOR. The sample clustered with the tumors of the HGNET-BCOR methylation class reinforcing the underlying assumption subsequently confirmed by RNA sequencing detecting an *EP300:BCOR* fusion (case previously reported in [14]). (**B**) Hematoxylin-phloxin-saffron stained sections of a tumor composed of uniform round embryonal cells showing Olig2 and synaptopysin expression consistent with the diagnosis of CNS NB-FOXR2 (case #40). Calibrated max-score was non-informative (0.31 for DMG H3K27 mutant). The sample clearly clustered with the tumors of the CNS NB-FOXR2 methylation class. Magnification x400.

In Figure 3, we illustrate two examples of representative cases for which the t-SNE analysis helped establish integrated diagnosis:

Case #16, for which the calibrated max-score was 0.57 for high grade neuroepithelial tumor with BCOR alteration (HGNET BCOR), clustered with the tumors of the HGNET BCOR methylation class. This result reinforced the underlying assumption that was confirmed by RNA sequencing that detected the *EP300:BCOR* fusion (Figure 3A, Table S1). These findings led us to definitively opt for the integrated diagnosis of HGNET BCOR (case previously reported in [14]).

Case #40, for which the calibrated max-score was 0.31 for diffuse midline glioma (DMG) H3K27 mutant, clearly clustered with the tumors of the CNS neuroblastoma with FOXR2 activation (CNS NB-FOXR2) methylation class, which was totally consistent with the clinical and histopathological findings, and corresponded to the diagnosis suggested by the experts at the national case conference (Figure 3B, Table S1). Additionally, the CNV analysis from the DNA methylation array showed a 1q gain. We thereby concluded with the integrated diagnosis of CNS NB-FOXR2.

### 3.4. Inference from Copy-Number Variation Data (CNV)

**Figure 4:**
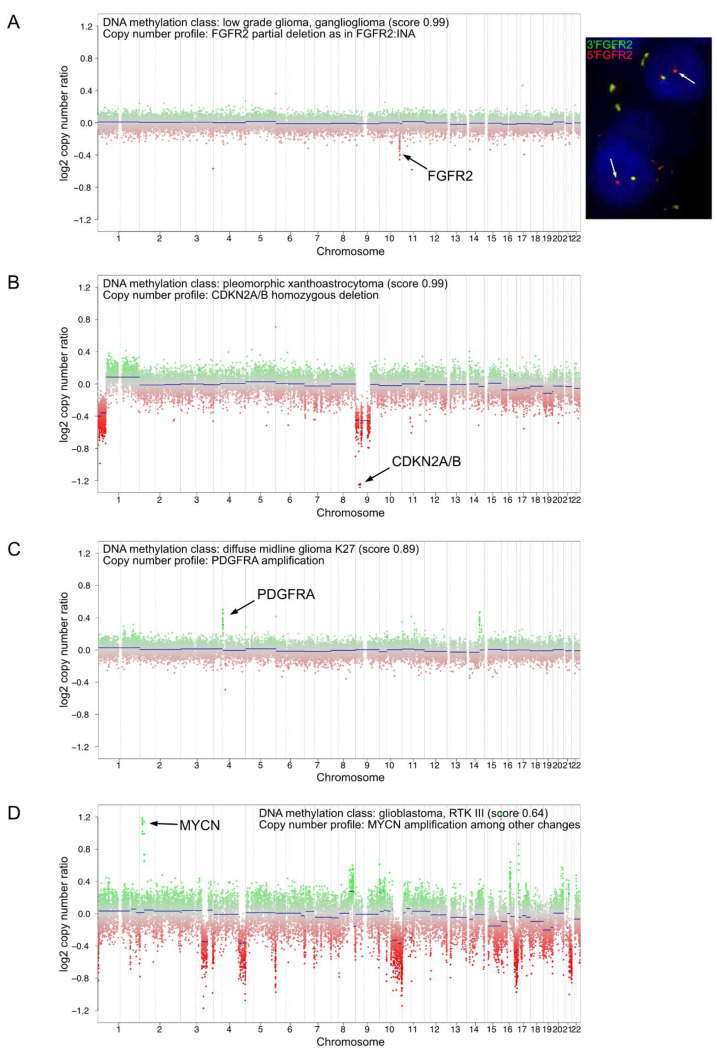
Examples of CNV plots from DNA methylation array showing relevant alterations for diagnosis, prognosis or precision medicine. (**A**) Example of a low-grade glioma (case #55) with a focal loss in chromosome 10q on the CNV plot (left plot) suggesting a *FGFR2* rearrangement subsequently confirmed by interphase FISH using a break-apart probe flanking FGFR2 (right picture). The white arrows show the FGFR2 locus rearranged. Magnification x600. (**B**) Example of a pleomorphic xanthoastrocytoma (case #50) with a CNV plot showing a homozygous deletion of *CDKN2A/B*. (**C**) Example of a diffuse midline glioma H3K27 mutant (case #62) with a CNV plot showing *PDGFRA* amplification. (**D**) Example of a glioblastoma RTKIII (case #53) with a CNV plot showing *MYCN* amplification. CNV plots depict chromosomes 1 to 22 with the p-arm (left) and the q-arm (right) separated by a dotted line.

As genome-wide DNA methylation array can also be used to generate broad copy-number data, we investigated the CNV profiles for every sample to collect further information on large-scale chromosomal losses and gains, focal gene amplifications or deletions. We considered CNV analysis to contribute to the final diagnosis independently from the “classifier” and the t-SNE analysis results. The CNV investigations evidenced alterations that helped us establish diagnoses and provided information for prognostication or information that could be interesting for precision medicine (Table S1). Focal gene deletions or amplifications were suggestive of gene fusion events, which were further confirmed by targeted method (FISH or targeted RNA sequencing) (Table S1). In Figure 4A, we illustrate one of these cases for which the CNV analysis detected a focal deletion including *FGFR2* gene. The FISH analysis using an FGFR2 break apart probe confirmed the presence of a rearrangement at this locus. Taking both morpho-molecular and methylation profiling data from this tumor, we refined the diagnosis to a polymorphous low-grade neuroepithelial tumor of the young (PLNTY) with *FGFR2* fusion. Additional illustrative cases with interesting CNV information that could impact decision-making are presented in Figure 4.

### 3.5. Final Data Integration Results

**Figure 5:**
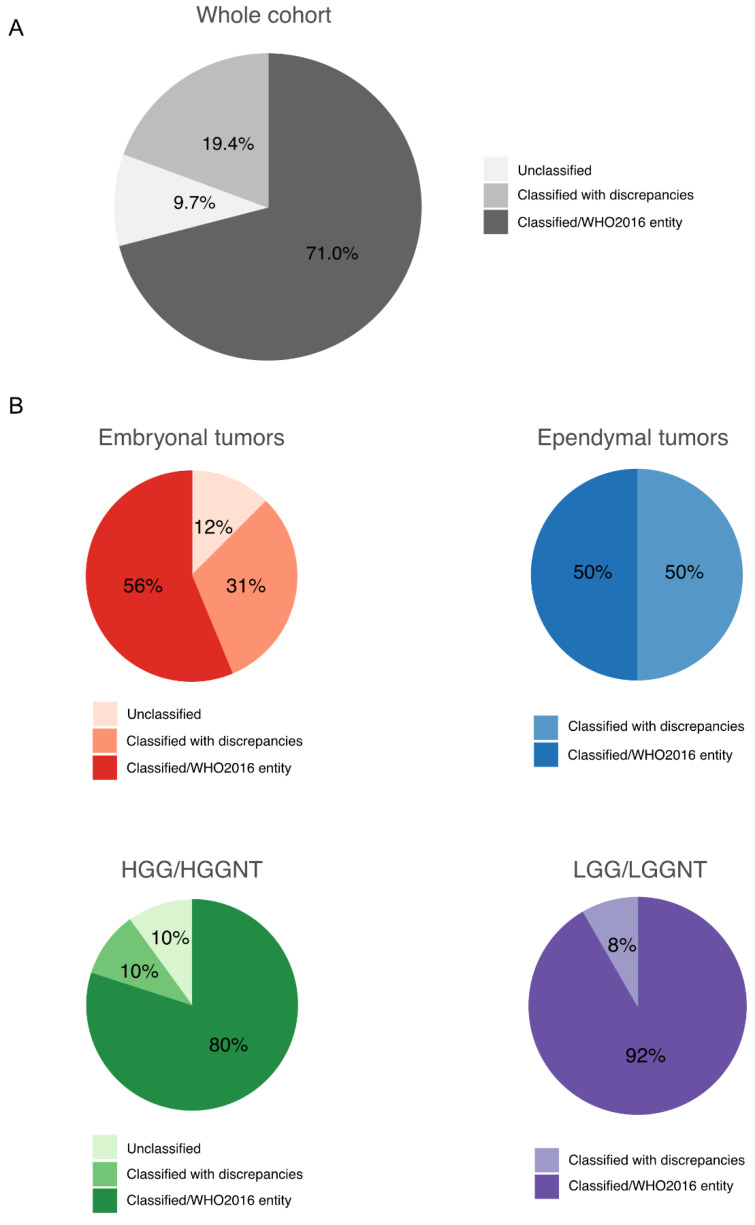
Final data integration results. Proportions of classified and unclassified tumors after integrating all data in the whole cohort (**A**) and in each category of tumors (**B**). HGG/HGGNT = high-grade glial/glioneuronal tumor. LGG/LGGNT = low-grade glial/glioneuronal tumor.

**Figure 6:**
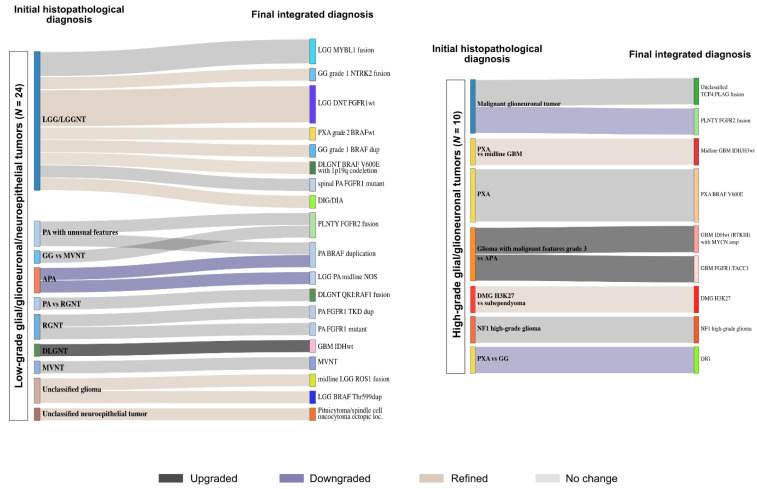
Establishing diagnoses in glial tumors. The integration of data from DNA methylation array to the initial histopathological diagnosis (left) helped establishing final diagnoses (right) in low-grade glial/glioneuronal tumors (left panel) and high-grade glial/glioneuronal tumors (right panel). WHO grading changes are indicated in dark grey (upgrading), blue (downgrading) and light brown (refined). LGG = low-grade glioma, LGGNT = low-grade glio-neuronal tumor, PA = pilocytic astrocytoma, GG = ganglioglioma, MVNT = multinodular and vacuolating neuronal tumor, APA = pilocytic astrocytoma with anaplastic features, RGNT = rosette forming glioneuronal tumor, DLGNT = diffuse leptomeningeal glioneuronal tumor, PXA = pleomorphic xanthoastrocytoma, DIG/DIA = desmoplastic infantile ganglioglioma/astrocytoma, PLNTY = polymorphous low-grade neuroepithelial tumor of the young, GBM = glioblastoma, DMG = diffuse midline glioma.

**Figure 7:**
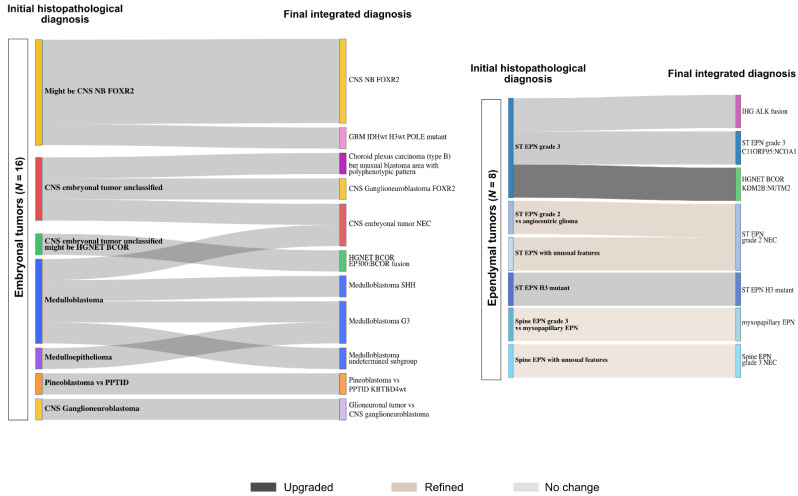
Establishing diagnoses in embryonal and ependymal tumors. The integration of data from DNA methylation array to the initial histopathological diagnosis (left) helped establishing final diagnoses (right) in embryonal tumors (left panel) and ependymal tumors (right panel). WHO grading changes are indicated in dark grey (upgrading), blue (downgrading) and light brown (refined). CNS NB FOXR2 = central nervous system neuroblastoma with FOXR2 activation, GBM = glioblastoma, HGNET-BCOR = high-grade neuroepithelial tumor with BCOR alteration, PPTID = pineal parenchymal tumor of intermediate differentiation, EPN = ependymoma, IHG = hemispheric infantile glioma, NEC = not elsewhere classified.

Finally, after 1 to 3 rounds of data integration, a diagnosis was established with certainty for 44 tumors (71%). For 12 additional cases (19.5%), we proposed a diagnosis but discrepancies persisted or unusual features were observed. Six tumors remained unclassified (Figure 5 and Table S1). Among the tumors initially diagnosed as embryonal tumors, 2 cases (12%) remained unclassified and discrepancies persisted in 5 cases (31%). Among the tumors initially diagnosed as ependymal tumors, a diagnosis was established in 4 cases (50%), including 2 tumors finally diagnosed as infantile hemispheric glioma and HGNET BCOR with a novel fusion (*KDM2B:NUTM2*). Diagnosis uncertainties were still observed in 4 tumors (50%). A diagnosis was established with certainty for, respectively, 80% and 92% of the tumors initially diagnosed as high-grade glial/glio-neuronal tumors (8/10) and low-grade glial/glio-neuronal tumors (22/24). One tumor harboring a novel fusion (*TCF4:PLAG*) was finally recorded as unclassified. The three tumors initially with no diagnostic hypothesis remained unclassified, including one harboring the recently reported novel fusion *EWSR1:PATZ1* [16]. The details about the changes are depicted in Figure 6 and Figure 7 and more details are provided in Table S1. Our work led us to upgrade 4 tumors and downgrade another 4. The grading was refined in 18 cases while it remained unchanged in 33 tumors (Figure 6 and Figure 7).

## 4. Discussion

The recent years have seen substantial transformation of CNS tumor nosology, adding complexity to their classification and thus requiring the development of the right tools to ascertain diagnoses in routine practice. The DNA methylation profiling approach has significantly contributed to this refinement and is increasingly becoming a tool of choice for routine diagnostic panel testing. Here we report the French RENOCLIP-LOC experience of the implementation of DNA methylation profiling into routine diagnostic practice for pediatric neuro-oncology.

Conversely to previously published works [10,11,12,13], we report an experience exclusively focused on challenging pediatric tumors selected after meticulous review by neuropathology experts in a real-time multilevel diagnostic process with a remarkable national organization, which is exceptional and rarely reported. Initially based on monthly “in-person” regional and national meetings, our organization has been turned upside down by the COVID-19 crisis that has boosted setting up of virtual meetings and led us to definitively adopt this technology due to streamlined logistics.

As previously reported, our results demonstrated the added value of this approach [10,11,12,13]. In our practice, DNA methylation profiling is proposed under collegial decisions and is exclusively restricted to challenging pediatric tumors with no consensus on the diagnosis, presenting unusual features or conflicting morpho-molecular data. Thus, the expected success rate was lower than in a cohort including no selected tumors since published data demonstrate that classification rates differ widely depending on the questions raised, the indication for methylation testing and the cohort analyzed [10]. Indeed, it has been demonstrated that DNA methylation profiling shows high performance in tumor subtyping (e.g., subtyping medulloblastomas) or in well-defined entities but is less successful when used to help establish a diagnosis for histopathologically challenging tumors with uncertain diagnoses [10]. In our study, despite the specificities of our cohort, we observed significant changes and DNA methylation was successful in a substantial number of cases that might impact treatment decisions. Even though our cohort consisted exclusively of tumors with uncertain diagnoses or unclassified tumors, we obtained a final diagnosis in 71% of the tumors, validating the relevance of the strategy of using this approach as a second line in the diagnostic process and not directly on the frontline. Our approach also confirms the previous compelling evidence of the impact on decision-making of incorporating DNA methylation profiling into routine testing in pediatric neuro-oncology. This strategy also allows us to use DNA methylation array in a cost-effective manner.

To optimize the use of the methylation assay and make the most of this approach, we attempted to thoroughly exploit the data obtained from the array to collect any information that may assist in decision making. Using the “classifier”, 53% of the diagnoses were confirmed, refined or amended while after combining the “classifier” results with t-SNE and CNV data alongside histopathologic examination, a diagnosis was proposed in 71% of the cases. CNV analysis allowed us to collect various relevant diagnostic, prognostic or theragnostic information. We identified focal copy-number changes suggesting fusion events that we could subsequently confirm using targeted methods such as FISH, which is particularly interesting for saving tumor tissue or when limited tissue is available for further analyses.

Despite all efforts, some tumors remained unclassified and/or with conflicting findings. Unclassified cases might correspond to rare entities that are not yet characterized. The integration of the data with clinic, imaging and histopathology and other molecular testing results represents the bulk of the work and is the key step.

The combination of histopathological examination with novel approaches such as DNA methylation profiling, alongside conventional molecular testing, is now primordial in the diagnostic process. Our work underlines that this integrated diagnostic process require to incorporate additional specific skills, especially in molecular biology and bioinformatics, and reflects the need to build multidisciplinary teams. A multilevel network can help to combine the knowledge and areas of expertise from the different centers, as well as reduce costs, technical constraints and laboratory requirement limitations.

## 5. Conclusions

Our cohort highlights the value of DNA methylation profiling in pediatric CNS tumors deemed histologically diagnostically difficult. Combined with traditional histopathological examination and conventional molecular testing, DNA methylation profiling is a powerful tool that provides multiple sources of information to help establish a diagnosis. Our results substantiate the strategy of using this approach as a second line in the diagnostic process of pediatric CNS tumors.

## Data Availability

Not applicable.

## Supplementary Materials

The following are available online at https://www.mdpi.com/2072-6694/13/6/1377/s1, Figure S1: Workflow of the RENOCLIP-LOC network, Table S1: Characteristics of the cohort.
