# Supplementary figures and images for: The Implementation of DNA Methylation Profiling into a Multistep Diagnostic Process in Pediatric Neuropathology: A 2-Year Real-World Experience by the French Neuropathology Network
Source: Cancers (Basel). 2021 Mar 18;13(6):1377. doi: 10.3390/cancers13061377 (PMC8003015; doi:10.3390/cancers13061377)

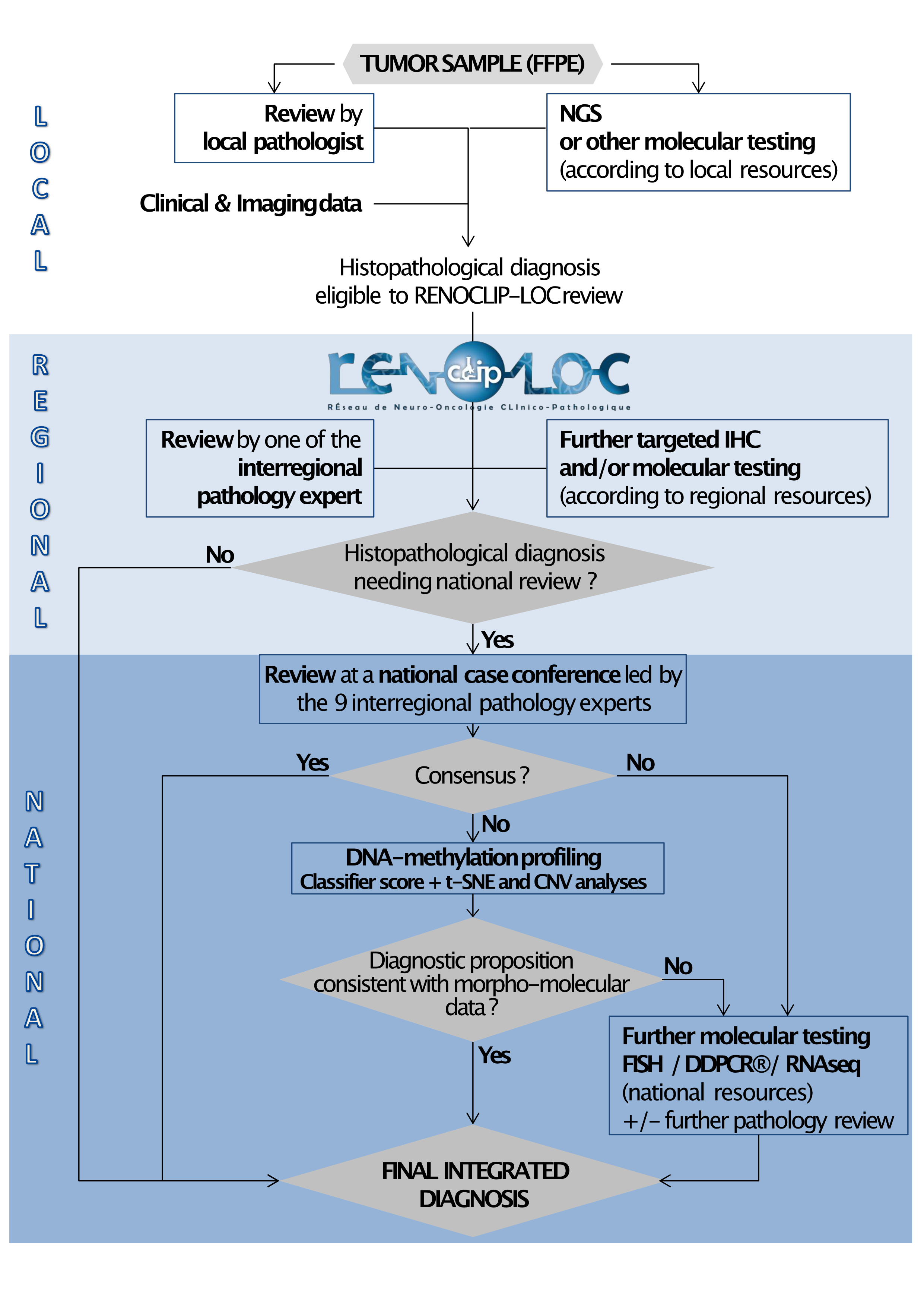

Supplement: Supplementary file 1 [file cancers-13-01377-s001.zip › Figure S1.tiff]
